# Supplementary material for: Alcohol consumption and health-related quality of life in the US during the COVID-19 pandemic: a US national survey
Source: J Patient Rep Outcomes. 2022 Oct 10;6:106. doi: 10.1186/s41687-022-00516-0 (PMC9550307; doi:10.1186/s41687-022-00516-0)
Supplement: Supplementary file 1 — Supplementary Material 1 [file 41687_2022_516_MOESM1_ESM.docx]

**Alcohol consumption and HRQOL in the US during the COVID-19 pandemic**

**Online and Telephone Survey Instrument**

**In general, would you say your health is^[[1]](#endnote-1)^**

Excellent

Very good

Good

Fair

Poor

**The following questions are about activities you might do during a typical day.**

**Does your health now limit you in these activities? If so, how much?**

- **moderate activities, such as moving a table, pushing a vacuum cleaner, bowling, or playing golf**

Yes, limited a lot

Yes, limited a little

No, not limited at all

- **climbing several flights of stairs**

Yes, limited a lot

Yes, limited a little

No, not limited at all

**During the past 4 weeks, how much of the time have you had any of the following problems with your work of other regular daily activities as a result of your physical health?**

- **Accomplished less than you would like?**

All of the time

Most of the time

Some of the time

A little of the time

None of the time

- **Were limited in the kind of work or other activities?**

All of the time

Most of the time

Some of the time

A little of the time

None of the time

**During the past 4 weeks, how much of the time have you had any of the following problems with your work of other regular daily activities as a result of any emotional problems (such as feeling depressed or anxious)?**

- **Accomplished less than you would like?**

All of the time

Most of the time

Some of the time

A little of the time

None of the time

- **Did work or other activities less carefully than usual?**

All of the time

Most of the time

Some of the time

A little of the time

None of the time

**During the past 4 weeks, how much did pain interfere with your normal work (including both work outside the home and housework)?**

Not at all

A little bit

Moderately

Quite a bit

Extremely

**These questions are about how you feel and how things have been with you during the past 4 weeks. For each question, please give the one answer that comes closest to the way you have been feeling. How much of the time during the past 4 weeks…**

- **Have you felt calm and peaceful?**

All of the time

Most of the time

Some of the time

A little of the time

None of the time

- **Did you have a lot of energy?**

All of the time

Most of the time

Some of the time

A little of the time

None of the time

- **Have you felt downhearted and depressed?**

All of the time

Most of the time

Some of the time

A little of the time

None of the time

All of the time

**During the past 4 weeks, how much of the time has your physical health or emotional problems interfered with your social activities (like visiting friends, relatives, etc.)?**

All of the time

Most of the time

Some of the time

A little of the time

None of the time

**The questions in this section are about your drinking of alcoholic beverages. When we ask how often you drink or how many drinks you consume, we are asking about your consumption of what are called standard drinks. One standard drink is any of these:**

- 12 ounce can of beer,
- 5 ounce glass of wine (table wine or fortified wine),
- A single wine cooler, hard seltzer, or malt liquor,
- a jigger or single shot of any hard liquor or spirits—like whiskey, gin, vodka, rum, or tequila.

**In the last 4 weeks, about how often did you have one or more standard drinks?**

Every day

Nearly every day

3 to 4 times a week

2 times a week

Once a week

2 to 3 times during the month

Once during the month

Never

**In the last 4 weeks, how many standard drinks did you usually have on days when you drank?**

[0-100] Drinks

**In the last 4 weeks, what was the largest number of standard drinks that you drank in a single day?**

[0-100] Drinks

**In the last 4 weeks, about how often did you drink your largest number of standard drinks that you told us about in the last question?**

Every day

Nearly every day

3 to 4 times a week

2 times a week

Once a week

2 to 3 times during the month

Once during the month

**In the last 4 weeks, about how often did you drink [male: FIVE; female: FOUR] OR MORE standard drinks within 2 hours?**

Every day

Nearly every day

3 to 4 times a week

2 times a week

Once a week

2 to 3 times during the month

Once during the month

**How has the amount you had to drink each week changed between now and before the pandemic began in early March, 2020?**

I’ve never been a drinker

I’m drinking a lot more than I did before

I’m drinking a little more than I did before

I’m drinking the same amount as I did before

I’m drinking a little less than I did before

I’m drinking a lot less than I did before

I’m not drinking now but did before

[*separate phrasing of responses for phone administration]*

*You’ve never been a drinker*

*You’re drinking a lot more than you did before*

*You’re drinking a little more than you did before*

*You’re drinking the same amount as you did before*

*You’re drinking a little less than you did before*

*You’re drinking a lot less than you did before*

*You’re not drinking now but did before*

**If you would like more information about consuming alcohol, please visit this website:** [**https://www.rethinkingdrinking.niaaa.nih.gov/**](https://www.rethinkingdrinking.niaaa.nih.gov/)

**These questions are about how your life has been impacted by the COVID-19 pandemic.**

**On a scale of 0 to 4 with 0 being not at all and 4 being extremely,**

**How much has your life been disrupted by COVID-19 related to…**

[presentation order randomized]

- **school changes or closures?**

Not at all

A little bit

Moderately

Quite a bit

Extremely

- **job loss or reduced hours?**

Not at all

A little bit

Moderately

Quite a bit

Extremely

- **social distancing and isolation?**

Not at all

A little bit

Moderately

Quite a bit

Extremely

- **loss of income?**

Not at all

A little bit

Moderately

Quite a bit

Extremely

**On the same scale of 0 to 4 with 0 being not at all and 4 being extremely,**

**How much has the covid-19 pandemic affected your own physical and emotional health?**

Not at all

A little bit

Moderately

Quite a bit

Extremely

**How much have you been affected by the way the COVID-19 pandemic has affected your relatives’ and friends’ physical and emotional health?**

Not at all

A little bit

Moderately

Quite a bit

Extremely

**Overall, how much has the COVID-19 pandemic impacted your life?**

Not at all

A little bit

Moderately

Quite a bit

Extremely

1. SF-12v2 copyright QualityMetric; used under license (https://www.qualitymetric.com/health-surveys-old/the-sf-12v2-health-survey/) [↑](#endnote-ref-1)
