# Supplementary material for: Alcohol consumption and health-related quality of life in the US during the COVID-19 pandemic: a US national survey
Source: J Patient Rep Outcomes. 2022 Oct 10;6:106. doi: 10.1186/s41687-022-00516-0 (PMC9550307; doi:10.1186/s41687-022-00516-0)
Supplement: Supplementary file 2 — Supplementary Material 2 [file 41687_2022_516_MOESM2_ESM.docx]

Abstract (word count = 348)

Background: Alcohol consumption has changed during the COVID-19 pandemic yet the impacts on alcohol-related outcomes, and specifically health-related quality of life, are not completely known. Our objective was to assess the association between alcohol consumption and health-related quality of life (HRQOL) during the COVID-19 pandemic.

Method: We conducted an on-line/telephone survey of three cross-sectional samples of US adults during a nine-month stretch of the pandemic, from August 2020 through April 2021, collecting data on drinking—current quantity/frequency and change since prior to pandemic, HRQOL (using the SF-6D), and perceived impact of the pandemic on respondents’ lives—overall impact and disruptions across various dimensions (job loss, school closures, social isolation, loss of income). We pooled the data from the three administrations and applied survey weights to reflect the US population. We described drinking behavior and pandemic impact, and regressed HRQOL on alcohol consumption risk level (per World Health Organization categories), change in drinking since pre-pandemic, and pandemic impact using weighted least squares, controlling for respondents’ demographic characteristics. We tested the significance of categorical variables using Wald tests at a p-value of 0.05.

Results: Among 3,125 respondents, weighted to reflect the US population, 68% reported drinking during the pandemic and 40% reported a change in drinking from pre-pandemic level (either increased or decreased). Mean HRQOL among our sample was 0.721 (SD 0.003). Any change in drinking from pre-pandemic level was independently associated with significantly lower HRQOL compared to never drinking (pre or during pandemic), from -0.0251 points for decreased/stopped drinking to -0.0406 points for increased drinking (combined levels’ Wald test *F*=10.62, p<0.0000). COVID-19 pandemic related impacts/disruptions were associated with HRQOL decrements ranging from -0.0834 to -0.1340 (Wald test *F*=64.34, p<0.0000)

Conclusions: The US population HRQOL was substantially lower during the pandemic than reported a decade earlier (mean = 0.79 in 2012-13). While pandemic-related impacts and disruptions may explain a large part of this decrement, changes in drinking—and the associated implications of such changes--might also play a role. Both individuals who reduced their drinking during the pandemic and those who increased consumption may be at risk of poor HRQOL.

Keywords: Utilities, SF-6D, HRQOL, alcohol, COVID-19

Plain English summary (144 words)

The COVID-19 pandemic has precipitated changes in drinking that may be positive or negative depending on who is affected and how. We conducted a survey of over 3,000 adults in the US during the middle of the pandemic to understand drinking and quality of life. We found that US adults’ quality of life was worse during the pandemic than in prior years, and people who either increased or decreased the amount that they drank were particularly worse-off. People who reported being severely impacted by the pandemic, however, were also much worse-off in terms of quality of life, and actually more so than people whose drinking habits changed. We need to pay attention to how drinking is connected to stressful events such as the pandemic, and make sure to attend to people who change their drinking in either direction as this may indicate underlying problems.

Background

Alcohol consumption changed during the COVID-19 pandemic, with reports documenting increased and decreased consumption, and substantial variability among population subgroups, types of drinkers, and types of drinking [1-3]. Possible explanations for such changes are varied, and include expanded access to alcohol due to relaxation of local laws (such as permitting “cocktails-to-go” and home delivery [4, 5]), drinking as a coping mechanism in response to pandemic-related stress and boredom [6], and changes in circumstances that enable or deter drinking—such as working from home or having children at home/home schooling [7].

Whether observed changes in alcohol consumption during the pandemic have impacted alcohol-related outcomes is as yet unknown. Research conducted prior to the pandemic has shown an association between alcohol consumption and health-related quality of life (HRQOL), with highest HRQOL among low-level drinkers (higher than non-drinkers/abstinence) and decreasing HRQOL as consumption increases [8]. We sought to understand the impact of pandemic-related changes in alcohol consumption on HRQOL by assessing the association between drinking and HRQOL in primary survey data from US adults.

Methods

Design: We conducted an on-line/phone survey of three cross-sectional samples of US adults during the pandemic—in August 2020, December 2020, and April 2021--to collect data on alcohol consumption, HRQOL, and perceived impact of the pandemic on respondents’ lives (see online supplemental information for survey instrument). The study was reviewed and considered exempt from human subjects review by the University of North Carolina Greensboro Institutional Review Board.

Sample: We used the NORC AmeriSpeak omnibus survey panel [9], a probability-based panel designed to be representative of the US household population. To develop the omnibus panel NORC randomly selects US households using area probability and address-based sampling, with a known, non-zero probability of selection from the NORC National Sample Frame. These sampled households are then contacted by US postal mail, telephone, and field interviewers (face to face) for enrollment into the panel. The panel provides sample coverage of approximately 97% of the US household population; those excluded include people with P.O. Box only addresses, some addresses not listed in the USPS Delivery Sequence File, and some newly constructed dwellings. While most AmeriSpeak households participate in surveys on-line, non-internet households can opt to participate by telephone, enabling both internet-connected and non-connected households to be included in the panel (approximately 85% participate on-line); NORC uses trained interviewers and established procedures to minimize divergence between modes of administration. The current panel totals nearly 50,000 members [9].

Omnibus panelists receive invitations bimonthly to respond to surveys on any topic or multiple topics; surveys are administered in English only. Surveys are “open” for responses for 3 days to allow for rapid delivery of results. We embedded our questions into survey versions distributed in our chosen months, each of which included multiple topics/questions besides ours. We limited our questions to respondents age 21 years and older as this is the minimum legal drinking age for most states.

Measures: Our embedded questions asked respondents to report their alcohol consumption quantity and frequency over the past 4 weeks based on similar questions included in the NESARC-III [10]; changes in their drinking since the start of the pandemic in March 2020, as drinking a lot more, a little more, the same amount, a little less, a lot less, or whether they were never a drinker (following others’ query types for pandemic-related drinking behavior [3]); whether they perceived disruption to their lives caused by pandemic-related school closures, job changes, income loss, and social isolation; whether they experienced pandemic-related effects on their physical/emotional health; and their overall assessment of how much the pandemic had impacted their life. We also included the SF-12v2 [11], a health status instrument from which the SF-6D can be derived; the SF-6D measures HRQOL on a 0-1 scale, with 1 representing the HRQOL associated with perfect health and 0 with being dead [12]. We used the 4-week recall format of the SF-12v2 to capture a more encompassing assessment of HRQOL than is reflected in the 1-week format and matched our questions about alcohol consumption quantity and frequency to the same 4-week time period. We pre-tested the survey questions with a convenience sample of six individuals and refined them for clarity and comprehension.

Analysis: We adjusted the sample to match US Census data on demographic characteristics using NORC-supplied survey weights [9]. We used descriptive statistics (means, proportions) to characterize our survey sample and responses to individual survey questions. We derived analytic variables as follows: we calculated SF-6D scores (i.e., HRQOL) from SF-12v2 responses using a published algorithm [12]; we used WHO alcohol risk consumption levels to designate five levels of consumption risk (no risk, low, moderate, high, and very high) using reported daily grams of ethanol consumed for men and women based on the drinks/day reported in the alcohol quantity and frequency questions, calculated from standard drink sizes [13]. WHO categories are no risk (mean consumption ≤ 1g daily), low risk (mean >1, ≤ 20g/day females; >1, ≤ 40g/day males), medium (> 20g, ≤ 40g/day females; > 40g, ≤ 60g/day males), high (> 40g, ≤ 60g/day females; > 60g, ≤ 100g/day males), and very high-risk consumption (> 60g/day females, > 100g/day males). We combined high and very high risk into one category due to small sample sizes of each. We collapsed reported change in drinking from pre-pandemic level from five to three levels: a lot more and a little more were combined into “increased drinking”, a lot less and a little less were “decreased drinking”, and no change was the third level; non-drinkers remained as a separate category. We pooled survey data across the three administrations for some analyses to reduce variability in the pandemic landscape over time and to minimize the impact of external/ environmental factors on HRQOL.

We used regression analysis to assess the association between HRQOL and alcohol consumption during the pandemic. We regressed SF-6D score (HRQOL) on WHO alcohol consumption risk level, reported change in alcohol consumption from pre-pandemic level, self-perceived overall pandemic impact, and demographic characteristics (NORC-collected and supplied: age, race/ethnicity, gender, education, marital status, current employment) using weighted least squares, and tested for joint significance of categorical variables using Wald tests (at p<0.05 level). We included pandemic impact and demographic characteristics as control variables, opting for overall pandemic impact rather than individual dimensions of pandemic-related impact for parsimony. All analyses were conducted using Stata 16.1 (StataCorp, LLC, College Station, TX). The dataset generated during and/or analyzed during the current study is available in the Harvard Dataverse repository [persistent web link to dataset to be added at time of publication].

Results

Our pooled sample totaled 3,125 respondents (approximately 1,000 respondents per administration). Participation rate = 20% of panel members recruited; repeat respondents across waves are estimated at no more than 1-2% of sample. Our population-weighted sample closely resembled the US population on demographic characteristics and alcohol consumption (Table 1). Forty-three percent of respondents were classified as WHO-defined low-risk alcohol consumption, and approximately 5% each as medium risk and high/very high risk; more women were in the no risk classification than men, fewer women were low risk than men, and approximately equal proportions of men and women were in the medium and high/very high risk classifications (results not shown). Approximately 29% of the sample reported no change in their drinking from pre-pandemic level, while approximately 16% reported an increase and 24% reported a decrease/cessation (approx. 32% reported never drinking pre or during pandemic though 47% were classified as non-drinkers within the past 4 week period by their reported drink consumption ). The mean SF-6D score was 0.721 (SD 0.003).

Approximately one-third of respondents reported the overall impact of the pandemic on their lives as extreme/quite a bit (36.04%), moderate (31.53%), or little/none (32.43%). Respondents reported the greatest disruption in their lives from social distancing and isolation, with nearly half (47.86%) reporting their lives were disrupted extremely or quite a bit; about one-fifth of respondents reported extreme or quite a bit of disruption from job loss (17.63%), loss of income (17.71%), and effects on own health (19.46%); about one-quarter (26.64%) reported this level of disruption from school changes or closures (Table 2). Half or more of respondents in our sample reported no disruption from school changes, job loss, and loss of income.

In our regression analysis (Table 3), any self-reported change in drinking from pre-pandemic level was independently associated with statistically significantly lower HRQOL compared to never drinking (pre or during pandemic): 0.0406 points lower for increased drinking (p<0.000) and 0.0251 points lower for decreased/stopped drinking (p=0.005); no change in drinking was associated with a 0.003-point higher HRQOL (non-significant individually: p=0.746; combined changes’ Wald test *F*=10.62, p<0.000). WHO alcohol consumption risk level was not significantly associated with HRQOL (Wald test *F*=2.20, p=0.0865). Reported extreme/quite a bit overall COVID-19 pandemic impact on respondents’ lives was associated with a statistically significant 0.1340 point lower HRQOL compared with no impact (p< 0.000), while moderate/little impact was significantly associated with 0.0834 point lower HRQOL (p<0.000; combined levels’ Wald test *F*=64.34, p<0 .000). All results were adjusted for respondent demographic characteristics; including an indicator variable for each survey administration in the model produced similar results (not shown).

Discussion

Our 2020-2021 survey of a US population-representative sample of 3,125 adults suggests that the mean health-related quality of life of the US adult population during the COVID-19 pandemic was substantially lower than it was in the past decade, currently at 0.72 compared with 0.79 in 2012-2013[8] and 0.80 in 2010-2015[14], all on the SF-6D scale (a difference of 0.03 to 0.04 is generally considered meaningful on this scale[15, 16]). Our survey results also show that self-reports of changes in alcohol consumption since pre-COVID-19 pandemic levels included increases, decreases, and stable consumption among respondents. We observed lower HRQOL associated with any reported change in drinking, either downward or upward, from -0.0251 to -0.0406, respectively. These decrements in HRQOL were far less than those associated with respondents’ reported impact of the pandemic on their lives, which ranged from -0.0834 to -0.1340, suggesting that the pandemic overall may have had a more important connection to individuals’ HRQOL than changes in drinking (although the two may be interconnected). These observed associations were independent of any association between alcohol consumption risk level and HRQOL as our models controlled for this factor.

Changes in alcohol consumption during the pandemic have been attributed to many factors,

varying by age and circumstance. For example, US college students’ COVID-19-related fears were associated with lower rates of risky drinking while the racial tensions in the US that coincided with the pandemic were associated with higher rates [17]; middle and older-age adults with depression, anxiety, or loneliness during the pandemic were more likely to report increased drinking [18]; similarly, adults with depression were at increased risk of binge drinking, as were people who spent more time under “shelter in place” orders [19]. Although our survey did not assess the reasons for changes in drinking from pre-pandemic levels, our results indicated that changes in drinking behavior corresponded with changes in HRQOL. We speculate that alcohol consumption could have been a reaction to negative repercussions of the pandemic, such as decreased drinking due to financial constraints or increased drinking due to pandemic-induced stress, or that changes in consumption could have been secondary to health effects of COVID-19, also in either direction. Our sample reported that social distancing and isolation significantly disrupted many people’s lives, as did school changes/closures, and there were widespread pandemic-related impacts on physical and emotional health. Health factors are known to diminish HRQOL [20], so if they also instigated changes in drinking there may be confounding in our results.

Of note, however, nearly 90% of our sample were at no or low-risk alcohol consumption levels, similar to US population findings from 2012-13 [8], with nearly 40% reporting a change in drinking behavior from pre to during the pandemic. While these measures reflect a different time period—alcohol consumption for the past 4 weeks and change in drinking behavior since March 2020— drinking behavior was perceived to fluctuate from pre-pandemic levels among our respondents despite overall risky drinking remaining approximately the same as in prior years. Drinking behavior may have varied over the course of the pandemic and/or been subject to recall bias, or reported changes may have remained within previous consumption risk levels—i.e., did not shift an individual to higher or lower risk. That individuals’ *perceived* changes in drinking patterns—as self-reported in the survey—were associated with lower HRQOL suggests that other factors may be at play than actual consumption, even though our analyses controlled for reported overall COVID-19 impact on respondents’ lives. It could be that external factors beyond what respondents perceived to be pandemic related impacts coincided with changes in drinking, which could explain the decreased HRQOL. For example, increased time spent with family members due to restrictions could have *increased* drinking and also decreased quality of life, and conversely increased time with children in the home due to school closures could have *decreased* drinking and also decreased quality of life; changes in job routines could have increased or decreased drinking and simultaneously decreased quality of life. There are a myriad of changes in life patterns and stressors that accompanied the early months of the pandemic all of which may have exacerbated problem drinking, and both the changes/stressors and problem drinking may have been associated with decreased HRQOL.[8] It is further possible that simply the perception of changed drinking patterns—that life had changed during the pandemic in this and many ways—could be reflected in lower HRQOL due to it being another involuntary change. Further research may elucidate the patterns that we found between drinking and HRQOL.

We could not control for additional confounders aside from those on which we collected data, and particularly other health conditions, so the underlying causes of the observed association remains an open question. Our results (though non-significant) for the HRQOL associated with risk consumption levels are consistent in direction with previous findings—low-risk consumption is associated with slightly higher HRQOL than no-risk consumption, and high/very high-risk consumption is associated with lower HRQOL than no-risk consumption. [8] Previous explanations for this pattern include that non-drinking, i.e., no-risk consumption, can often be a result of health conditions or medications that interact with alcohol, so the “better” HRQOL of low-risk consumption versus non-drinking is a function of better health rather than more drinking.

As with all survey data, our results come with some caveats: budgetary constraints limited our sample size and prevented us from looking at potentially important heterogeneity among subgroups. We attempted to protect against selection bias in our survey sample in multiple ways: by using a nationally representative panel, embedding our questions within a larger set of unrelated topics to minimize interest/disinterest in our particular topic, utilizing both online and telephone survey administration to include respondents without access to or comfort with the internet, and using US population weighting in our analysis. Our confidence is increased by our regression coefficients being in the expected direction for the COVID-19 impact variables and demographic characteristics, but selection bias is still a potential concern in our results and appropriate caution in interpretation is warranted. In addition, the SF-6D is but one measure of HRQOL that, although potentially more accurate than alternatives for mild conditions [21], could miss important aspects of the relationship between alcohol consumption, the COVID-19 pandemic, and HRQOL. Confirmation using other HRQOL metrics is warranted to confirm our results. Our data are self-reported and there is a risk of recall or social desirability bias. We used similar questions to those used in the NESARC-III, an established alcohol study [10], but our data collection mode and resources were more constrained than those employed by a federally-administered population survey and are correspondingly less robust to error. Finally, we acknowledge that there may have been other, unmeasured factors that influenced the association between alcohol consumption and HRQOL during the pandemic that impacted our results.

While diminished HRQOL during the pandemic and particularly diminished HRQOL associated with changes in alcohol consumption are concerning, our survey results capture a short time frame since the start of the pandemic in March 2020 and downstream impacts of the pandemic and drinking are yet unknown. It appears that overall alcohol consumption risk level has not substantially changed on a population level despite numerous reports of changes in consumption, ours among them. Future research will be needed to understand the long-term impact of alcohol consumption during this event, particularly among population subgroups that may be more susceptible to hazardous drinking and the associated health outcomes, as well as among low-risk drinkers for whom potential drinking-related outcomes may be overwhelmed by the overall impact of the pandemic on their lives. Long-term health effects of excessive drinking may take years to manifest, whereas the short-term benefits of drinking as a coping mechanism may be obvious immediately. Other consequences of changes in alcohol consumption that are important in policy decisions are beyond the scope of HRQOL metrics, such as motor vehicle fatalities and lost productivity. These may also be obscured during the pandemic through other societal changes. As we emerge from the pandemic additional data may shed light on the complex relationship between drinking and HRQOL under changing circumstances, and particularly during crises.

Despite the need for additional data and research, our results could suggest that pandemic-related changes in drinking may be the result of, rather than the cause of, observed reductions in HRQOL at this time. Although this conclusion is somewhat speculative, it is consistent with our finding that both self-perceived increases and decreases in consumption were related to lower HRQOL, and with our finding that the underlying relationships between consumption risk levels and HRQOL is qualitatively similar to the relationships found a decade ago. Possible mechanisms explaining increases or decreases in drinking include physical and mental health changes and solitude [22], all of which may have an impact on HRQOL. If directionality of changes in consumption is unimportant in the potential HRQOL association, as our results suggest, alcohol interventions in the upcoming time might better target pandemic-related impacts than consumption targets, as both individuals reducing drinking may be at similar risk of poor HRQOL as well as those in higher consumption categories.

Conclusions

Drinking patterns have changed during the COVID-19 pandemic and diminishing HRQOL has been observed. The relationship between these phenomena is unclear but data suggest that there may be a connection: the pandemic may have led to health effects or a social environment that affected drinking and consequently HRQOL, or drinking may have changed as a direct result of the pandemic, as a coping mechanism or due to environmental changes, resulting in diminished HRQOL. It will be important to disentangle these effects over time to fully understand the relationship among the contributing factors, and to discern whether population mean HRQOL returns to pre-pandemic levels in the coming years. The survey data presented in this paper add one piece to this evolving puzzle.


Table 1. Sample characteristics-unweighted n and US population-weighted proportions for each survey administration and combined samples

|  | Dec. 2020  (n=1046) | April 2021  (n=998) | Aug. 2021  (n=1081) | Total  (n=3125) |
| --- | --- | --- | --- | --- |
| Female  Age (years)  21-45  46-65  Over 65  Race/Ethnicity  White, non-Hispanic  Black, non-Hispanic  Hispanic, any race  2+ races, non-Hispanic  Asian, non-Hispanic  Other race, non-Hispanic  Education  No HS Diploma  HS Grad or Equivalent  Some College/Associate Degree  Bachelor’s Degree or Higher  Household Income (annual)  Less than $30,000  $30,001 - $60,000  $60,001- $100,000  $100,000 +  Marital Status  Married  Widowed  Divorced  Separated  Never Married  Live-in Partner  Current Employment  Employed  Self-Employed  Temporary Layoff  Seeking Work  Retired  Disabled  Not Working, Other  WHO alcohol consumption risk level  No risk  Low  Medium  High/very high^2^  Reported change in drinking from March 2020 to current^1^  Never been drinker  Increased  No change  Decreased/stopped    SF-6D score (mean, SD) | 511 (51.67%)  499 (46.68%)  315 (32.19%)  232 (21.13%)  679 (63.33%)  137 (11.87%)  165 (16.25%)  23 (2.06%)  29 (4.60%)  13 (2.88%)  46 (8.77%)  166 (26.9%)  400 (26.68%)  434 (37.65%)  260 (28.86%)  303 (28.39%)  264 (22.02%)  219 (20.73%)  518 (47.84%)  46 (4.89%)  116 (10.12%)  43 (4.91%)  242 (24.71%)  81 (7.53%)  544 (47.84%)  90 (8.62%)  9 (1.36%)  43 (4.43%)  207 (19.42%)  77 (9.00%)  76 (9.32%)  487^1^ (47.96%)  499 (39.23%)  25 (4.93%)  24 (6.85%)  330 (34.88%)  173 (17.53%)  314 (28.16%)  216 (19.43%)  0.718 (0.004) | 505 (51.67%)  451 (43.99%)  321 (33.96%)  226 (22.06%)  648 (63.33%)  102 (11.87%)  153 (16.25%)  31 (1.4%)  37 (5.42%)  27 (1.73%)  32 (8.82%)  151 (27.71%)  432 (26.97%)  383 (36.50%)  206 (22.49%)  306 (28.36%)  252 (25.76%)  234 (23.39%)  499 (48.19%)  40 (4.33%)  128 (14.38%)  47 (6.02%)  230 (22.47%)  54 (4.60%)  530 (49.18%)  102 (10.91%)  7 0.86%)  47 (6.44%)  204 (19.66%)  51 (6.64%)  57 (6.31%)  475 (48.72%)  414 (40.7%)  55 (5.03%)  54 (5.55%)  274 (28.63%)  127 (15.06%)  314 (28.31%)  276 (27.99%)  0.737 (0.004) | 615 (51.78%)  460 (45.22%)  366 (33.84%)  255 (20.95%)  758 (62.90%)  125 (11.94%)  109 (16.47%)  42 (2.87%)  33 (4.99%)  14 (0.83%)  38 (8.51%)  178 (28.37%)  442 (26.33%)  423 (36.79%)  264 (26.82%)  296 (26.97%)  294 (25.35%)  227 (20.86%)  565 (47.77%)  51 (4.02%)  111 (10.62%)  49 (4.75%)  248 (27.32%)  57 (5.53%)  547 (49.59%)  94 (11.30%)  9 (0.52%)  47 (6.28%)  242 (19.12%)  77 (7.11%)  65 (6.07%)  510 (47.93%)  432 (38.78%)  74 (7.37%)  65 (5.92%)  337 (31.38%)  160 (14.90%)  325 (30.26%)  252 (23.46%)  0.727 (0.004) | 1,631 (51.71%)  1,410 (45.31%)  1,002 (33.33%)  713 (21.36%)  2,085 (63.18%)  364 (11.89%)  427 (16.33%)  96 (2.13%)  99 (5.00%)  54 (1.47%)  1,472 (48.36%)  1,345 (41.99%)  154 (5.07%)  143 (4.58%)  730 (26.12%)  905 (27.89%)  810 (24.37%)  680 (21.62%)  1,582 (47.93%)  137 (4.41%)  355 (11.65%)  139 (5.21%)  720 (24.90%)  192 (5.90%)  1,621 (48.88%)  286 (10.28%)  25 (0.91%)  137 (5.71%)  653 (19.40%)  205 (7.59%)  198 (7.23%)  1,472 (48.39%)  1,345 (41.99%)  154 (5.07%)  143 (4.58%)  941 (31.74%)  460 (15.81%)  953 (28.67%)  744 (23.81%)   - 1. (0.003) |

1. 11 total missing responses in WHO alcohol consumption risk level and 21 in Reported change in drinking from March 2020 to current
2. Categories combined due to small sample size

HS=High school; SD=standard deviation


Table 2. Survey responses on COVID-19 impact questions--unweighted n and US populaton weighted proportions (n=3125)

|  | n (%) | | | | |
| --- | --- | --- | --- | --- | --- |
| Level of disruption to life due to: | Extreme | Quite a bit | Moderate | A little bit | Not at all |
| school changes or closures | 431  (14.04%) | 385 (12.60%) | 383  (12.61%) | 291  (9.63%) | 1,601 (51.12%) |
| job loss or reduced hours | 276  (9.02%) | 242 (8.61%) | 310  (10.99%) | 352  (10.80%) | 1,913 (60.58%) |
| social distancing and isolation | 691  (22.89%) | 806 (24.97%) | 769  (24.17%) | 523  (17.07%) | 314  (10.90%) |
| loss of income | 251  (8.70%) | 258 (9.01%) | 352  (11.88%) | 494  (16.89%) | 1,746 (53.52%) |
| Impact of pandemic on own physical and emotional health | 191  (6.53%) | 399 (12.93%) | 811  (25.47%) | 1,066 (34.14%) | 638  (20.93%) |
| Overall impact of pandemic on own life (Total)  Wave 1 only (n=1046)  Wave 2 only (n=998)  Wave 3 only (n=1081) | 354  (12.44%)  136  (13.87%)  107  (11.25%)  111 (12.19%) | 778 (23.60%)  303 (28.66%)  237 (23.19%)  238 (19.14%) | 1,023 (31.53%)  327 (29.54%)  318 (31.82%)  378 (33.16%) | 754  (25.41%)  210 (21.91%)  263  (26.88%)  281 (27.38%) | 193  (7.02%)  55  (6.02%)  72  (6.85%)  66  (8.12%) |


Table 3. Weighted least squares regression model predicting SF-6D score from WHO alcohol consumption risk level, reported change in drinking during pandemic, and self-reported overall COVID-19 pandemic impact on own life (n=3,125)

|  | Coefficient (SE) | p-value | Wald test  *F* (p-value) |
| --- | --- | --- | --- |
| WHO alcohol consumption risk level (no risk=reference group) |  |  |  |
| Low | 0.0119 (0.0079) | 0.132 | 2.20 (0.0865) |
| Moderate | -0.0151 (0.0144) | 0.292 |  |
| High/very high | 0.0163 (0.0166) | 0.326 |  |
| Reported change in drinking (never-drinker=reference group) |  |  |  |
| Increased | -0.0406 (0.0115) | 0.000 | 10.62 (0.0000) |
| No change | 0.0030 (0.0093) | 0.746 |  |
| Decreased/stopped | -0.0251 (0.0100) | 0.005 |  |
| Overall COVID-19 impact on own life (no impact = reference group) |  |  |  |
| A little bit/moderate | -0.0834 (0.0128) | 0.000 | 64.34 (0.0000) |
| Quite a bit/extreme | -0.1340 (0.0133) | 0.000 |  |
| Age (21- 45 = reference group)  46 – 65 years old  Over 65 years old | 0.0280 (0.0073)  0.0471 (0.0118) | 0.000  0.000 | 10.60 (0.0000) |
| Race/Ethnicity (White, non-Hispanic = reference group  Black, non-Hispanic  Hispanic  Other/2+/Asian, non-Hispanic | 0.0142 (0.0091)  -0.0070 (0.0089)  -0.0123 (0.0099) | 0.116  0.433  0.220 | 1.84 (0.1370) |
| Gender (Male = reference group)  Female | -0.0167 (0.0060) | 0.005 | 7.72 (0.0055) |
| Education (No HS diploma = reference group)  HS Grad or Equivalent  Some College/Associate Degree  Bachelor’s Degree or Higher | 0.0194 (0.0149)  0.0216 (0.0143)  0.0486 (0.0147) | 0.193  0.129  0.001 | 6.39 (0.0000) |
| Marital Status (Married = reference group)  Widowed  Divorced  Separated  Never Married  Live in partner | -0.0083 (0.0140)  -0.0094 (0.0098)  -0.0236 (0.0121)  -0.0224 (0.0077)  -0.0053 (0.0116) | 0.551  0.336  0.051  0.004  0.645 | 2.17 (0.0552) |
| Current Employment (Employed = reference group)  Self-Employed  Temporary Layoff  Seeking Work  Retired  Disabled  Not Working, Other | 0.0064 (0.0110)  -0.0379 (0.0233)  -0.0505 (0.0145)  -0.0346 (0.0113)  -0.1462 (0.0115)  -0.0157 (0.0117) | 0.561  0.104  0.001  0.002  0.000  0.181 | 29.56 (0.0000) |

SE= standard error

References

1. Barbosa C, Cowell AJ, Dowd WN. Alcohol Consumption in Response to the COVID-19 Pandemic in the United States. J Addict Med. 2021;15(4):341-4.

2. Pollard MS, Tucker JS, Green HD, Jr. Changes in Adult Alcohol Use and Consequences During the COVID-19 Pandemic in the US. JAMA Netw Open. 2020;3(9):e2022942.

3. Schmidt RA, Genois R, Jin J, Vigo D, Rehm J, Rush B. The early impact of COVID-19 on the incidence, prevalence, and severity of alcohol use and other drugs: A systematic review. Drug Alcohol Depend. 2021;228:109065.

4. U.S. Census Bureau. Retail Sales: Beer, Wine, and Liquor Stores [MRTSSM4453USN]: FRED, Federal Reserve Bank of St. Louis; 2020 [Available from: <https://fred.stlouisfed.org/series/MRTSSM4453USN>.

5. The Nielsen Company. Rebalancing the ‘COVID-19 Effect’ on alcohol sales 2020 [updated May 7, 2020. Available from: <https://www.nielsen.com/us/en/insights/article/2020/rebalancing-the-covid-19-effect-onalcohol-sales/>.

6. Grossman ER, Benjamin-Neelon SE, Sonnenschein S. Alcohol Consumption during the COVID-19 Pandemic: A Cross-Sectional Survey of US Adults. Int J Environ Res Public Health. 2020;17(24).

7. Chartier KG, Karriker-Jaffe KJ. Commentary on Nordeck et al.: Disruption of social roles and daily routines in the initial phase of the COVID-19 pandemic may explain changes in alcohol use frequency. Addiction. 2021;117(2):341-42.

8. Barbosa C, Bray JW, Dowd WN, Barnosky A, Wittenberg E. SF-6D utility scores for alcohol use disorder status and alcohol consumption risk levels in the US population. Addiction. 2021;116(5):1034-42.

9. NORC. NORC AmeriSpeak Omnibus 2021 [Available from: <https://amerispeak.norc.org/our-capabilities/Pages/AmeriSpeak-Omnibus.aspx>.

10. Grant B, Chu A, Sigman R, Amsbury M, Kali J, Sugawara Y, et al. Source and Accuracy Statement: National Epidemiologic Survey on Alcohol and Related Conditions-III (NESARC-III). Rockville, MD: NIAAA; 2012.

11. Ware J, Kosinski M, Turner-Bowker D, Gandek B. How to score version 2 of the SF-12® health survey (with a supplement documenting version 1). Lincoln, RI

QualityMetric Incorporated; 2002.

12. Brazier JE, Roberts J. The estimation of a preference-based measure of health from the SF-12. Med Care. 2004;42(9):851-9.

13. World Health Organization. International guide for monitoring alcohol consumption and related harm. Doc No: WHO/MSD/MSB/00.4. Geneva, Switzerland: World Health Organization; 2000.

14. Song HJ, Heo JH, Wilson DL, Shao H, Park H. A National Catalog of Mapped Short-Form Six-Dimension Utility Scores for Chronic Conditions in the United States From 2010 to 2015. Value Health. 2022;epub.

15. Luo N, Johnson J, Coons SJ. Using instrument-defined health state transitions to estimate minimally important differences for four preference-based health-related quality of life instruments. Med Care. 2010;48(4):365-71.

16. Walters SJ, Brazier JE. Comparison of the minimally important difference for two health state utility measures: EQ-5D and SF-6D. Qual Life Res. 2005;14(6):1523-32.

17. Lechner WV, Laurene KR, Patel S, Anderson M, Grega C, Kenne DR. Changes in alcohol use as a function of psychological distress and social support following COVID-19 related University closings. Addict Behav. 2020;110:106527.

18. Eastman MR, Finlay JM, Kobayashi LC. Alcohol Use and Mental Health among Older American Adults during the Early Months of the COVID-19 Pandemic. Int J Environ Res Public Health. 2021;18(8).

19. Weerakoon SM, Jetelina KK, Knell G. Longer time spent at home during COVID-19 pandemic is associated with binge drinking among US adults. Am J Drug Alcohol Abuse. 2021;47(1):98-106.

20. Wittenberg E, Barbosa C, Hein R, Hudson E, Thornburg B, Bray JW. Health-related quality of life of alcohol use disorder with co-occurring conditions in the US population. Drug Alcohol Depend. 2021;221:108558.

21. Kontodimopoulos N, Pappa E, Papadopoulos AA, Tountas Y, Niakas D. Comparing SF-6D and EQ-5D utilities across groups differing in health status. Qual Life Res. 2009;18(1):87-97.

22. Roberts A, Rogers J, Mason R, Siriwardena A, Hogue T, Whitley G, et al. Alcohol and other substance use during the COVID-19 pandemic: a systematic review. Drug Alcohol Depend. 2021;229:109150.

Declarations

Ethics approval: The study was reviewed and considered exempt from human subjects review by The University of North Carolina Greensboro Institutional Review Board

Consent for publication: Not applicable

Availability of data and materials: Survey instrument is available in the online supplementary information at [URL to be added]. The dataset generated during and/or analyzed during the current study are available in the Harvard Dataverse repository, [PERSISTENT WEB LINK TO DATASETS to be added at time of publication].

Competing interests: The authors declare that they have no competing interests.

Funding: [included in title page to preserve blinding]

Authors’ contributions: [included in title page to preserve blinding]

Acknowledgements: We are grateful for Achele Agada’s assistance in developing and pretesting the survey instrument and for the comments of two anonymous reviewers
